# Phthalate-associated hypertension in premature infants: a prospective mechanistic cohort study

**DOI:** 10.1007/s00467-019-04244-4

**Published:** 2019-04-26

**Authors:** Randall Jenkins, Shane Tackitt, Ladawna Gievers, Sandra Iragorri, Kylie Sage, Tonya Cornwall, Declan O’Riordan, Jennifer Merchant, David Rozansky

**Affiliations:** 1 Department of Pediatrics, Oregon Health & Science University, 707 SW Gaines Road, Mail Code CDRC-P, Portland, OR 97239 USA; 2 HCA Medical City Weatherford, Weatherford, TX USA; 3 Biostatistics and Design Program, School of Public Health, Oregon Health & Science University, Portland, OR USA; 4 St. Luke’s Regional Medical Center, Boise, ID USA

**Keywords:** Hypertension, Neonatal, Phthalates, Blood pressure

## Abstract

**Background:**

Phthalates are associated with increased blood pressure in children. Large exposures to di-(2-ethylhexyl) phthalate (DEHP) among premature infants have been a cause for concern.

**Methods:**

We conducted a prospective observational cohort study to determine if DEHP exposures are related to systolic blood pressure (SBP) in premature infants, and if this exposure is associated with activation of the mineralocorticoid receptor (MR). Infants were monitored longitudinally for 8 months from birth. Those who developed idiopathic hypertension were compared with normotensive infants for DEHP exposures. Appearance of urinary metabolites after exposure was documented. Linear regression evaluated the relationship between DEHP exposures and SBP index and whether urinary cortisol/cortisone ratio (a surrogate marker for 11β-HSD2 activity) mediated those relationships. Urinary exosomes were quantified for sodium transporter/channel expression and interrogated against SBP index.

**Results:**

Eighteen patients met the study criteria, nine developed transient idiopathic hypertension at a postmenstrual age of 40.6 ± 3.4 weeks. The presence of urinary DEHP metabolites was associated with prior IV and respiratory tubing DEHP exposures (*p* < 0.05). Both IV and respiratory DEHP exposures were greater in hypertensive infants (*p* < 0.05). SBP index was related to DEHP exposure from IV fluid (*p* = 0.018), but not respiratory DEHP. Urinary cortisol/cortisone ratio was related to IV DEHP and SBP index (*p* < 0.05). Sodium transporter/channel expression was also related to SBP index (*p* < 0.05).

**Conclusions:**

Increased blood pressure and hypertension in premature infants are associated with postnatal DEHP exposure. The mechanism of action appears to be activation of the MR through inhibition of 11β-HSD2.

**Electronic supplementary material:**

The online version of this article (10.1007/s00467-019-04244-4) contains supplementary material, which is available to authorized users.

## Introduction

The only phthalate approved by the U.S. Food and Drug Administration for use in medical supplies is di-(2-ethylhexyl) phthalate (DEHP), a compound commonly added to polyvinyl chloride (PVC) to soften plastic. DEHP has been identified in a variety of hospital devices including a selection of intravenous (IV) fluid bags and some respiratory equipment [1–5]. Inherent use of such devices in a neonatal intensive care unit (NICU) may expose small premature infants to relatively high levels of DEHP, exceeding typical adult exposures by several orders of magnitude [1, 3, 6].

In 2013, with a report from Trasande et al. and later corroborated by others, phthalates surfaced as a cause of increased blood pressure in children and adults [7–10]. At present, the mechanism of this increase remains unclear. However, only a few years earlier, Zhao et al. pointed toward a possible mechanism by showing that certain DEHP metabolites cause sodium retention due to a licorice-like action inhibiting the enzyme11β-hydroxysteroid dehydrogenase type 2 (11β-HSD2) [11].

In 2017, our group reported on a large cohort of 97 premature infants with idiopathic low-renin hypertension, some of whom had chronic lung disease [12]. Al Awad et al. recently reported on a homogenous group of premature infants with low-renin hypertension, all of whom had chronic lung disease [13]. Hypertensive patients in these two studies all shared certain similarities including near-universal low or undetectable plasma renin activity (PRA), presentation of high blood pressure around 35–40 weeks postmenstrual age (PMA), and an excellent response to treatment with spironolactone. These similarities suggest the possibility of a unifying mechanism for the observed hypertension involving sodium transport in the distal nephron.

When considered in concert, the three concepts outlined above—large exposure to DEHP, a potential mechanism of sodium retention tied to DEHP, and cohorts of premature infants with low-renin hypertension—provide a basis for the hypothesis that DEHP increases blood pressure and may cause hypertension in premature infants. This current report tests this hypothesis both by comparing DEHP exposure between hypertensive and normotensive premature infants, as well as by examining the linear relationship between DEHP exposure and blood pressure in the overall cohort. Further, we investigate the infants’ absorption of DEHP based on the detection of toxic urine metabolites, including the principal metabolite, mono-(2-ethylhexyl) phthalate (MEHP) [14–16]. Lastly, we explore a plausible mechanism associated with idiopathic low-renin hypertension in premature infants by evaluating for MEHP-inhibited 11β-HSD2 and consequential activation of mineralocorticoid receptor pathways.

## Methods

A prospective observational study was conducted at two medical centers with approvals from the Institutional Review Boards and informed consent given for all participants. We recorded DEHP exposures and blood pressures in premature infants from birth for at least 6 months or until resolution of hypertension off medication occurred, whichever came last. Based on our prior study, we would expect no new cases of idiopathic hypertension after 6 months PMA, nor would we anticipate the ongoing need for anti-hypertensive medications beyond 8 months PMA [12]. Inclusion and exclusion criteria (Table 1) were designed to avoid bias related to clinical factors that might affect blood pressure. Study funding limited initial enrollment to 20 subjects, after which an analysis of the data was performed and presented herein. Two subjects were recruited monthly into the study until enrollment was complete. Although there were perhaps as many as 600 total potential premature infants to enroll, only 2–4 met the study criteria at each of our bi-monthly recruiting days. The infants with the lowest gestational age were approached first to minimize the number of outpatient visits required.

**Table 1:** Inclusion and exclusion criteria for study

Inclusion criteria	Exclusion criteria	Additional exclusion criteria at time of diagnosis of hypertension
Gestation age < 37 weeks	Secondary hypertension^a^	Receiving IV fluid or sodium supplementation
Subject resides within 90 miles of center	Chronic kidney disease—any stage	Receiving diuretic or sympathomimetic agents
Age 2 weeks or less at entry^b^	Congenital renal abnormality^c^	Acute kidney injury^d^
	Foster care, or surrogate birth	Patent ductus arteriosus (current)

^a^Chronic lung disease was not considered a cause of secondary hypertension

^b^This criteria was added after the first subject enrolled for the purpose of collecting a single urine sample for phthalate metabolites early in life when phthalate exposure was more likely to be occur

^c^Mild hydronephrosis and mild nephrocalcinosis were not considered exclusions

^d^Acute kidney injury was defined as serum creatinine > 0.6 mg/dL or urine output less than 1.0 mL/kg/h

The *principal statistical comparisons* between normotensive and hypertensive groups for DEHP exposures, blood pressure, and diagnostics were planned to be performed at the time of diagnosis of hypertension (and a corresponding PMA for normotensive infants). The diagnosis of hypertension was anticipated to be near 40 weeks PMA based on our prior work [12].

### Study procedures

Table 2 describes the timeline and plan for study procedures. At enrollment, birthweight, gestational age at birth, and receipt of antenatal steroids were recorded, as these factors have been shown to be associated with (but not causative for) hypertension in premature infants [17–21]. Urine for sodium channel expression (described below) and a one-time urine sample for urine phthalate metabolites were the only diagnostic tests obtained at enrollment. The presence of DEHP exposure (defined below) for the 48 h prior to this one-time urine sampling was recorded since humans excrete DEHP into urine 12 to 48 h after DEHP exposure [22]. The presence of any DEHP exposure during the 48-h window was compared with the presence of any DEHP urine metabolites, including MEHP and two oxidative metabolites: mono-(2-ethyl-5-hydroxyhexyl) phthalate (MEHHP) and mono-(2-ethyl-5-oxohexyl) phthalate (MEOHP). To include the measurements of MEHHP and MEOHP results in an increase in the detection sensitivity for DEHP exposure as compared to MEHP measurement alone [3], urine phthalate metabolites were measured in a commercial lab using high performance liquid chromatography with tandem mass spectrometry (LC-MS/MS).

**Table 2:** Timing of study procedures

Study Enrollment	Monitoring visits	Main Comparison	Post-hypertension visits	Study End
First month of life	Every 4 weeks from enrollment through 4 months	At diagnosis of hypertension or 40 weeks PMA (if hypertensive)	Every four weeks after diagnosis of hypertension	Resolution of hypertension-at least six months of age
SBP index	SBP index^a^	SBP index	SBP index	SBP index
Urinary ENaC, pNCC	Urinary ENaC, pNCC	U. ENaC, pNCC, cortisol/cortisone	Urinary ENaC, pNCC	U. ENaC, pNCC, cortisol/cortisone
Informed consent, demographics and risk factors for hypertension	Hypertension determination	PRA, serum sodium, potassium, creatinine, and aldosterone	Resolution of hypertension determination	Resolution of hypertension determination
Urine phthalate analysis (one-time)	Interval DEHP exposures	Cumulative DEHP exposures from birth		Medication history
Inclusion/exclusion		Inclusion/exclusion		

*PMA* postmenstrual age, *ENaC* epithelial sodium channel, *pNCC* phosphorylated (activated) sodium chloride cotransporter, *DEHP* di-(2-ethylhexyl phthalate), *SBP* systolic blood pressure, *PRA* plasma renin activity

^a^SBP index (SBP/SBP 95th percentile) was determined at each visit and every 2 weeks for inpatients

Between enrollment and the time of the *principal comparison* (at hypertension diagnosis), every other week systolic blood pressure (SBP) and all DEHP exposures (defined below) were recorded. Urinary exosome expression of the epithelial sodium channel (ENaC) and phosphorylated (activated) sodium chloride cotransporter (pNCC) were obtained every 4 weeks. These two sodium channels are upregulated by activation of the mineralocorticoid receptor (MR) [23, 24]. The procedure for isolation of urinary exosomes was described by Van der Lubbe et al., with a modification such that urine and reagent volumes were scaled down by two-thirds [25]. Activities were measured with Western Blot methodology using antibodies to the gamma subunit of ENaC, pNCC, and CD9 (a marker of exosome material), and were expressed as a ratio of transporter expression with CD9 expression. Concise methods for exosome preparation and Western analysis are available in the Appendix.

Diagnostic tests reflecting sodium metabolism were obtained at the time of diagnosis of hypertension or at 40 weeks PMA for normotensive patients—the *principal comparison* timeframe. These tests, which were all obtained prior to initiating anti-hypertensive therapy (for hypertensive infants), were as follows: serum sodium, potassium, creatinine, aldosterone, plasma renin activity (PRA), ENaC, pNCC, and urine cortisol-to-cortisone ratio, a surrogate for 11β-HSD2 activity. Hypertensive and normotensive groups were compared for these tests as well as for blood pressure and DEHP exposures.

### Quantitation of DEHP exposures

Equipment was evaluated for the presence of DEHP at both hospitals based on product labeling.

Di-(2-ethylhexyl) pthalate was found in selected IV fluid bags (but not in parenteral nutrition bags) and in most respiratory-related tubing (except low-flow nasal cannulas). Since product labels list the presence but not the amount of DEHP in the product, cumulative exposures to DEHP were quantified in aggregate by the following methodology: The volume (mL) of IV fluid administered to the infant from DEHP-containing IV bags quantified the IV exposure and respiratory tubing exposure was quantified by the number of days the patient was connected to any respiratory tubing containing DEHP.

### Blood pressure measurement and cohort assignment

Systolic blood pressure was measured by nurses using the oscillometric method for infants while in the NICU and by a single experienced physician using the auscultatory method on the right arm when seen in the outpatient clinical setting. The two oscillometric devices used were from Philips Medical Systems, BG Eindhoven, The Netherlands, and from SpaceLabs Inc., Redmond, Washington, U.S.A. For these blood pressure measurements, there was no specification as to which extremity was used for testing. Bedside, nurses were trained in the use of appropriate-sized blood pressure cuffs. Diastolic blood pressures were not used due to reported concern with accuracy of auscultatory diastolic blood pressure measurement in this age group [12]. Outpatient visits occurred every 4 weeks through 4 months of age (or resolution of hypertension), with a final visit at resolution of hypertension but no earlier than 6 months of age. If SBP exceeded the gestational age–adjusted 95th percentile, the infant was asked to return within 7 days for a blood pressure recheck.

Assignment to either the hypertensive or normotensive cohort was based on blood pressure criteria. Infants met the criteria for inclusion in the hypertensive group if their mean daily SBP (3 or more measurements per day) exceeded the 95th percentile for at least three sequential days while in the NICU or three sequential visits for outpatients. The “day of diagnosis” for hypertensive patients was set as the first date that the diagnosis of hypertension was recorded in the medical record. For three cases where the “day of diagnosis” of hypertension was not clearly established in the medical record, the “day of diagnosis” was set as the date the patient first met the above blood pressure criteria. The SBP 95th percentiles used for this study originated from the normative data compiled by Dionne et al., which provides blood pressure norms adjusted by PMA for premature infants [26]. Because SBP varies greatly with PMA, SBP index (SBP/SBP 95th percentile) was calculated and used to represent systolic blood pressure relative to the PMA-adjusted 95th percentile for SBP.

### Statistical analysis

A non-parametric, two-sided Wilcoxon rank sum test compared differences between the normotensive and hypertensive groups for continuous variables. Continuity correction was applied when needed. Pearson’s chi-squared testing was used to compare categorical variables. When expected values for chi-squared testing were < 5, an N-1Chi-squared test was used [27]. Correlations between DEHP exposures and risk factors of neonatal hypertension were calculated using a Pearson’s correlation. Statistical analyses for this report were performed using R version 3.3.2 and Stata version 14.2 (R Core Team 2016; Stata Corp. 2015).

We examined the relationship between cumulative DEHP exposures and the SBP index on the day of diagnosis for hypertensive infants and at the 40 weeks PMA visit for normotensive infants using linear regressions. First, we modeled cumulative IV DEHP and respiratory DEHP exposures. Next, a 4-step mediation analysis was performed to understand whether the relationship between IV DEHP exposure and SBP index is mediated by the urine cortisol-to-cortisone ratio. If the magnitude of the coefficient greatly decreased when the cortisol-to-cortisone ratio was added to the model, one would conclude that the exposure and cortisol-to-cortisone ratios were inter-related [28]. Finally, to test the hypothesis that activation of the MR was the putative mechanism of blood pressure elevations seen in study infants, we performed linear regression between expression of the two MR–regulated renal sodium channel transporters, (ENaC and pNCC), and SBP index at the same principal comparison timeframe.

## Results

Of the 26 patients approached for participation, 20 enrolled in the study. One patient unexpectedly died in the first month of life for reasons unrelated to blood pressure and another failed to return for study visits prior to the 40-week PMA visit. This left 18 patients available for analysis. No secondary hypertension occurred in this cohort, and no other patients met exclusion criteria.

Nine patients developed hypertension at a mean PMA of 40.6 ± 3.4 weeks. Three of these nine fulfilled criteria and began treatment for hypertension while still in the NICU. Two cases experienced mild hypertension while in the NICU and did not receive anti-hypertensive medication. Four of these nine patients met hypertensive criteria as an outpatient. Only three of these four received anti-hypertensive therapy. The duration of treatment for the six infants treated for hypertension was 14.2 ± 4.9 weeks. Hypertension only lasted 2.6 ± 0.7 weeks in the three untreated hypertensive infants. All treated infants received spironolactone monotherapy with SBP successfully dropping below the adjusted 95th percentile within 1 week. Spironolactone in treated patients was discontinued successfully between 48 and 64 weeks PMA. All patients were off treatment at the study’s end, with normal blood pressure not different from normotensive patients (*p* < 0.05).

**Fig. 1:**
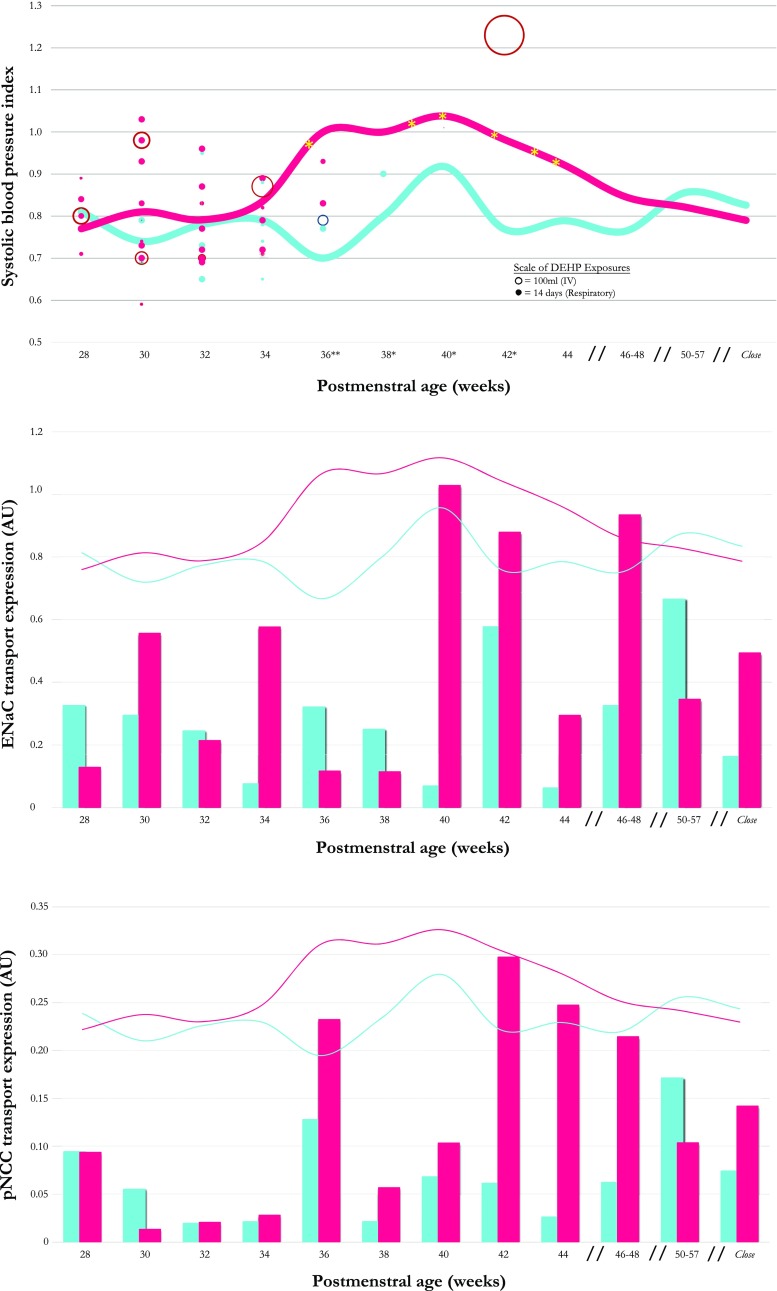
**a**. Systolic blood pressure index and DEHP exposures in normotensive (cyan) versus hypertensive infants (magenta) are shown in 2-week intervals from 28 weeks postmenstrual age to the last study visit (approximately 8 months of age). Individual patient 2-week interval DEHP exposures are shown for both intravenous (open circles) and respiratory-related DEHP exposures (solid circles). Each point represents the time-specific SBP index of an individual subject, with the size of the point or circle adjusted according to the magnitude of the exposure. Since the relationship between IV and respiratory DEHP exposures is unknown, the scales used for the two different DEHP exposure types are not related. Yellow asterisks within the magenta line mark the individual start of spironolactone treatment for the six treated hypertensive patients. Significant differences in SBP index between groups are noted on the X axis as follows: *p < 0.05, **p < 0.001. **b**. SBP index (lines) from **a** and mean ENaC activity (columns) in normotensive (cyan) versus hypertensive infants (magenta) is shown in 2-week intervals from 28 weeks PMA to the last study visit. **c**. SBP index (lines) from **a** and mean pNCC activity (columns) in normotensive (cyan) versus hypertensive infants (magenta) from 28 weeks PMA to the last study visit. SBP, systolic blood pressure; PMA, postmenstrual age; DEHP (di-(2-ethylhexyl) phthalate; ENaC, epithelial sodium channel; pNCC, phosphorylated (activated) sodium chloride cotransporter

Figure 1 provides a visual depiction of SBP index, intravenous, and respiratory-related DEHP exposures, and sodium transporter expression throughout the study period. Figure 1a depicts the average SBP index in hypertensive versus normotensive patients in relation to the type of DEHP exposure, whether respiratory or intravenous. On average, SBP for the hypertensive group did not increase until after 34 weeks PMA, with the SBP index peaking at 40 weeks PMA. Average group SBP normalized as infants began treatment with spironolactone. Normotensive patients showed a blunted peak of SBP index at 40 weeks PMA, but did not achieve levels consistent with systolic hypertension. Most exposures to either intravenous or respiratory DEHP in hypertensive patients occurred early in life, beginning before any rise in SBP index, mostly complete by 36 weeks PMA. The median time between the subjects’ last DEHP exposure and diagnosis of hypertension was 3.57 weeks. Figure 1 b and c show the urinary exosomal expression of ENaC and pNCC for hypertensive and normotensive groups reported in 2-week intervals of postmenstrual age. Increased ENaC expression in the hypertensive group coincided with the peak SBP index around 40 weeks PMA. More interestingly, pNCC urinary exosomal expression in the hypertensive group was similar to that in the normotensive group through 34 weeks PMA. After 34 weeks PMA, pNCC expression was consistently higher in the hypertensive group compared to the normotensive group for the time when SBP index was higher in the hypertensive group.

Next, we move from the overview to our *principal statistical comparison* between the hypertensive group (at the diagnosis of hypertension), and the normotensive group (at 40 weeks PMA) for demographics, DEHP exposures, and risk factors for hypertension, which are shown in Table 3. Both intravenous and respiratory cumulative DEHP exposures were greater in the hypertensive as compared to the normotensive group (*p* = 0.029, *p* = 0.041 respectively). Individual patient data for exposures and onset of hypertension are available in the Appendix. Gender, race, birthweight, receipt of antenatal steroids, and diagnosis of chronic lung disease did not differ significantly between the groups. Gestational age was lower in the hypertensive group, (*p* = 0.012). In addition, the length of stay for the initial NICU hospitalization was longer in the hypertensive group (*p* = 0.027).

**Table 3:** Characteristics and comparisons between hypertensive and normotensive groups

	Hypertensive group	Normotensive group	
	*n* = 9	*n* = 9	*p* value
Clinical characteristics			
Gender: male (#)	4	7	0.136^a^
Race: White, non-Hispanic (#)	7	6	0.492^b^
Antenatal steroids received (#)	9	8	1.000^b^
Chronic lung disease (#)	2	1	0.515^a^
Birth weight (kg)	1.21 ± 0.64	1.72 ± 0.69	0.136^d^
Gestational age at birth (weeks)	28.5 ± 2.2	32.6 ± 2.9	0.012^c^
Length of stay in neonatal unit (days)^g^	22 (36)	91 (27)	0.027^d^
Comparisons			
Between groups at onset of hypertension (40 weeks CGA for normotensive patients)			
Time frame by CGA of comparison (weeks)	40.6 ± 3.4	40.3 ± 3.3	0.930^c^
Cumulative IV fluid DEHP exposure from birth to main comparison (mL)^g^	201.3 (291.7)	0 (0)	0.029^c^
Cumulative respiratory DEHP exposure from birth to main comparison (days)^e,g^	45 (38)	7 (7)	0.041^c^
SBP index (percentage of age-adjusted 95th percentile)	1.11 ± 0.10	0.86 ± 0.08	< 0.001^d^
Plasma renin activity below 2.0 ng/mL/h (#)	9	2	0.003^a^
Serum sodium (meq/L)	138.9 ± 2.8	140.0 ± 2.6	0.447^d^
Serum potassium (meq/L)	5.2 ± 0.6	5.3 ± 1.0	0.781^d^
Serum creatinine (mg/dL)	0.27 ± 0.06	0.29 ± 0.05	0.576^d^
Serum aldosterone (ng/dL)^g^	55.4 (31.8)	20.9 (25.3)	0.164^d^
Urine cortisol-to-cortisone ratio	0.07 ± 0.02	0.03 ± 0.01	0.020^d^
pNCC protein expression^f,g^	0.402 (0.127)	0.086 (0.105)	0.042^d^
ENaC protein expression^f,g^	0.723 (0.316)	0.279 (0.243)	0.031^d^
Between groups at study close			
Time frame by postnatal (chronologic) age (weeks)	34.0 ± 8.4	36.5 ± 10.3	0.401^d^
Systolic blood pressure (mmHg)	83.22 ± 6.87	86.78 ± 4.47	0.211^d^

Continuous variables are shown as mean (±SD); *CGA* corrected gestational age, *PDA* patent ductus arteriosus, *DEHP* di-(2-ethylhexyl) phthalate, *SBP* systolic blood pressure, *ENaC* epithelial sodium channel, *pNCC* phosphorylated (activated) sodium chloride cotransporter

^a^N-1 chi-squared test

^b^Pearson’s chi-squared test with Yates’s continuity correction

^c^Wilcoxon rank sum test with continuity correction

^d^Wilcoxon rank sum test

^e^This variable includes days of mechanical ventilation, CPAP, high-flow nasal cannula, non-invasive positive pressure ventilation, but not low-flow nasal cannula

^f^Sodium channel transport expression is expressed as activity per CD9 activity

^g^Data are presented as median (IQR) due to their non-normal distribution

All hypertensive patients had PRA levels that were below 2.0 ng/mL/h, compared to two of nine normotensive infants (*p* = 0.003). The common lower limit of normal for PRA in children is 2.0 ng/mL/h. At the 40-week PMA principal comparison point, urinary cortisol-to-cortisone ratios were significantly higher in hypertensive infants as compared with normotensive infants (*p* = 0.02). Both ENaC and pNCC, markers of MR activation, were significantly higher in hypertensive patients (*p* = 0.042, *p* = 0.031).

**Fig. 2:**
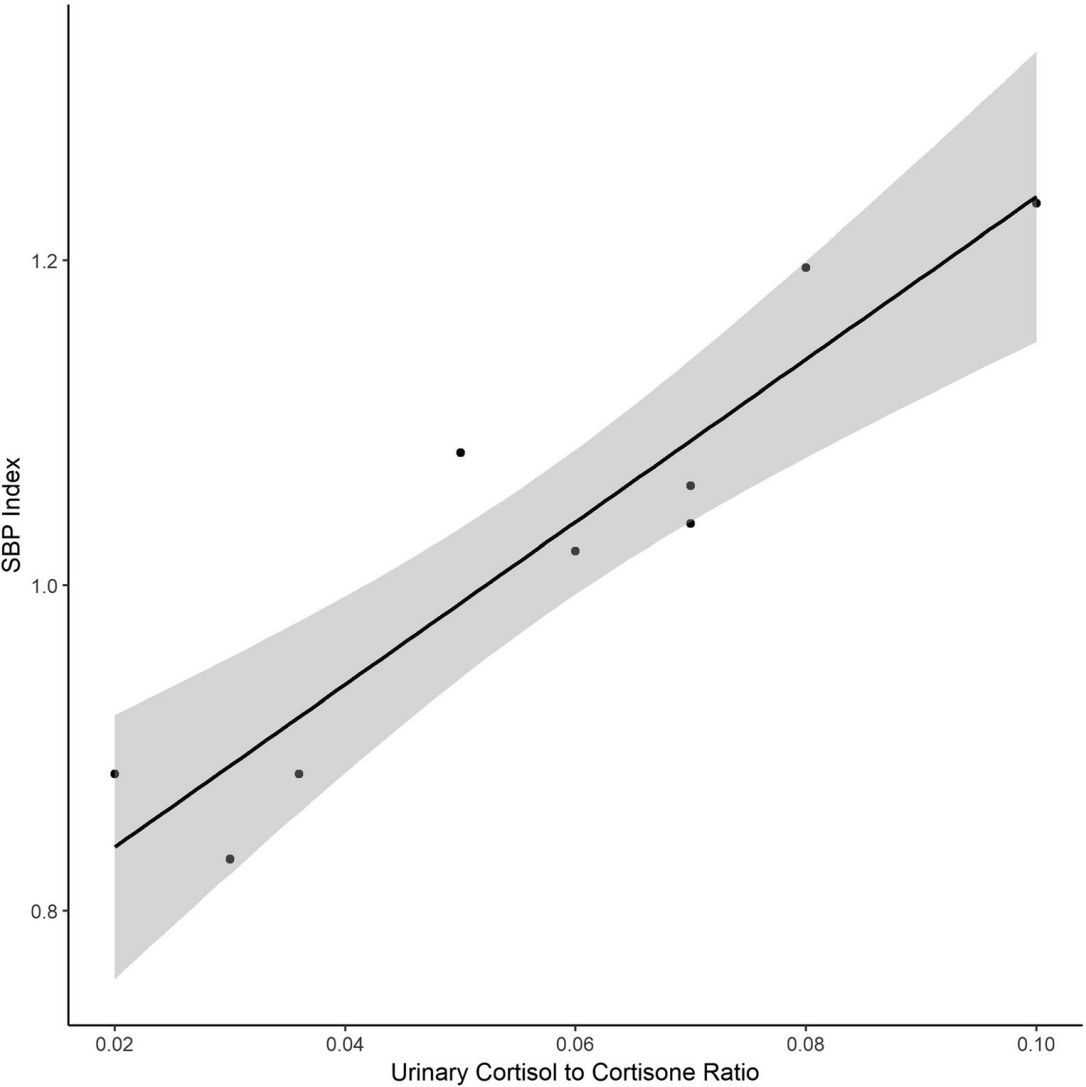
Linear regression for urinary cortisol-to-cortisone ratio as a predictor of systolic blood pressure (SBP) index at the time of diagnosis of hypertension (or 40 weeks postmenstrual age for normotensive infants (*p* < 0.001). The SBP index is the ratio of SBB with the 95th percentile for SBP adjusted for postmenstrual age. Depicted is the linear regression. SBP, systolic blood pressure

Correlations between exposures and previously reported risk factors of hypertension in premature infants are shown in Table 4. Gestational age, birthweight, and length of stay are all correlated with cumulative respiratory DEHP exposure but not with IV DEHP exposure. Linear regression analysis demonstrated that SBP index at the principal comparison (onset of hypertension) was associated with cumulative IV DEHP exposure, but not respiratory DEHP exposure (Table 5). The urinary cortisol-to-cortisone ratio was predicted by intravenous DEHP exposure, and was highly predictive of SBP index. Figure 2 shows the close relationship between urinary cortisol-to-cortisone ratio and SBP index at onset of hypertension (or 40 weeks PMA for normotensive infants). In a multivariable model including IV DEHP exposure and the cortisol-to-cortisone ratio, the magnitude of the IV DEHP exposures effect on SBP index attenuates and approaches zero—evidence that the urinary cortisol-to-cortisone ratio mediates the relationship between DEHP exposure and SBP index (Table 6). Finally, both pNCC and ENaC were also predictive of SBP index at the diagnosis of hypertension (or 40 weeks PMA for normotensive infants) (Table 7).

**Table 4:** Pearson’s correlation coefficients for relationships between DEHP exposures and risk factors of hypertension in premature infants

	IV DEHP	*p*	Respiratory DEHP	*p*
Gestational age	− 0.135	0.59	− 0.865	< 0.001
Birthweight	0.047	0.85	− 0.767	< 0.001
Length of stay	0.134	0.59	0.849	< 0.001

**Table 5:** Linear model results for the relationship between DEHP exposures and systolic blood pressure (SBP) index at the diagnosis of hypertension (or 40 weeks postmenstrual age for normotensive infants)

Independent variable	Dependent variable	*ß* coefficient	(95% confidence interval)	*p*
Univariate model				
IV DEHP exposure (mL)	SBP index	0.0004	0.0001–0.0008	0.018
Respiratory DEHP exposure (days)	SBP index	0.0007	− 0.0020-0.0034	0.587

*DEHP* di-(2-ethylhexyl) phthalate, *IV* intravenous, *SBP* systolic blood pressure

**Table 6:** Mediation analysis to evaluate urine cortisol-to-cortisone ratio as a mediator of the IV DEHP effect on systolic blood pressure index at the diagnosis of hypertension (or 40 weeks PMA for normotensive infants). The magnitude of the coefficient drops toward zero when urine cortisol-to-cortisone ratio is added to the model, suggesting the latter ratio mediates the effect of IV DEHP exposure on SBP index

Independent variable	Dependent variable	*ß* coefficient	(95% confidence interval)	*p*
Univariate model				
IV DEHP exposure (mL)	Cortisol-to-cortisone ratio	0.0001	1.64e − 06−0.0001	0.046
Urine cortisol-to-cortisone ratio	SBP index	5.0389	3.2058–6.8721	< 0.001
IV DEHP exposure (mL)	SBP index	0.0004	0.0001–0.0008	0.018
Multivariable model including IV DEHP exposure and cortisol-to-cortisone ratio				
IV DEHP exposure (mL	SBP index	− 0.0001	− 0.0003–0.0002	0.571
Urine cortisol-to-cortisone ratio	SBP index	5.4849	2.7859–9.1839	0.003

*DEHP* di-(2-ethylhexyl) phthalate, *SBP* systolic blood pressure, *IV* intravenous, *PMA* postmenstrual age

**Table 7:** Linear regression model results for the relationship between sodium channel expression and SBP index at the diagnosis of hypertension (or 40 weeks postmenstrual age for normotensive infants)

Independent variable	Dependent variable	*ß* coefficient	(95% confidence interval)	*p*
Univariate model				
pNCC	SBP index	0.7025	0.3115–1.0934	0.003^a^
ENaC	SBP index	0.1955	0.0205–0.3704	0.032^a^

*DEHP* di-(2-ethylhexyl) phthalate, *IV* intravenous, *SBP* systolic blood pressure, *pNCC* phosphorylated (activated) sodium chloride cotransporter, *ENaC* epithelial sodium channel

^a^*p* < 0.05

The results for the DEHP urine metabolite testing are shown in Table 8. One sample was obtained in 14 of 20 enrolled infants—all at the study initiation visit (when blood pressure was normal in all). DEHP metabolites were present for six of ten infants with DEHP exposures documented during this 48 h window pre-sample. By N-1 Chi-square analysis, the presence of DEHP metabolites was related to the prior 48-h IV DEHP exposure (*p* = 0.030), as well as prior respiratory DEHP exposure (*p* = 0.033). In contrast, when no DEHP exposure occurred during this 2-day period, no patient was found with detectable urinary DEHP metabolites.

**Table 8:** Presence or absence of urinary DEHP metabolites as detected from a single urine sample at study enrollment as compared with presence or absence of DEHP exposures during the prior 48 h

Subject (#)^a^	Respiratory DEHP exposures (days)	IV DEHP exposures (mL)	MEHHP (ng/mL) (mcg/g creatinine)	MEOHP (ng/mL) (mcg/g creatinine)	MEHP (ng/mL) (mcg/g creatinine)
N1^d^	2 (bubble CPAP)	0	Assay failed	1600 12,000	650 4600
N2^b^	0	0	< LOD	< LOD	< LOD
N3^b^	0	0	< LOD	< LOD	< LOD
N4^b^	0	0	< LOD	< LOD	< LOD
N5^d^	2 (Hi-flow and ventilator)	9	Assay failed	< LOD No urine creatinine	30 No urine creatinine
N6^c^	2 (Hi-flow)	0	Assay failed	Assay failed	< LOD
N7^e^	NA	NA	Not done	Not done	Not done
N8^e^	NA	NA	Not done	Not done	Not done
N9^e^	NA	NA	Not done	Not done	Not done
D1^d^	2 (Ventilator)	10	Assay failed	69 420	32 200
D2^e^	NA	NA	Not done	Not done	Not done
H1^e^	NA	NA	Not done	Not done	Not done
H2^d^	2 (NIPPV)	0	Assay failed	Assay failed	35 240
H3^c^	2 (NIPPV)	0	< LOD	< LOD	< LOD
H4^b^	0	0	< LOD	< LOD	< LOD
H5^d^	2 (Hi-flow)	49	45 400	25 220	< LOD
H6^c^	2 (mask CPAP)	0	< LOD	< LOD	< LOD
H7^c^	2 (mask CPAP)	12	< LOD	< LOD	< LOD
H8^d^	2 (NIPPV)	24	49 400	18 150	< LOD
H9^e^	NA	NA	Not done	Not done	Not done

*DEHP* di-(2-ethylhexyl) phthalate, *MEHHP* mono-(2-ethyl-5-hydroxyhexyl) phthalate, *MEOHP* mono-(2-ethyl-5-oxohexyl) phthalate, *MEHP* mono-(2-ethylhexyl) phthalate, *CPAP* continuous positive airway pressure, *NIPPV* non-invasive positive pressure ventilation, *LOD* level of detection

^a^Urine samples (one each) were obtained on 14 subjects including 1 dropout (D), 6 normotensive (N), and 8 hypertensive (H) subjects

^b^Infants with no exposures and no detectable phthalate metabolites

^c^Infants with DEHP exposures (3 respiratory and 1 combined) and no detectable phthalate metabolites

^d^Infants with DEHP exposures (2 respiratory and 4 combined) who had detectable urine phthalate metabolites

^e^Infants where no sampling was done

## Discussion

This study presents novel evidence that DEHP exposure is associated with systolic blood pressure increases in premature infants, including hypertension with larger exposures. The case for intravenous DEHP exposure increasing blood pressure is particularly strong given the following four findings: the linear relationship with SBP index, mediation of this relationship by the cortisol-to-cortisone ratio, and mechanistic links to MR activation.

The relationship between respiratory DEHP exposure and SBP is more complex. Although associated with hypertension in the studied cohort, no linear relationship was observed between respiratory DEHP exposure and SBP index, at least at the time of the diagnosis of hypertension. The inability to quantify respiratory DEHP exposure with precision confounded the ability to determine such a relationship. In addition, by virtue of the presence of DEHP in most respiratory equipment, respiratory DEHP exposure remains integrally linked with chronic lung disease and other markers of prematurity. This observation raises the question whether respiratory DEHP has a causal role in hypertension, or is merely just another marker of chronic lung disease and other risk factors related to extreme prematurity. A recent report of Stroustrup et al. showing premature infants exposed to respiratory support had significantly higher levels of DEHP urine metabolites compared to unexposed infants suggests the need for an in-depth look at the impact of respiratory DEHP on neonatal blood pressure [5].

The delay between DEHP exposures and peak blood pressure is notable, especially for intravenous DEHP exposures. One possible explanation for the apparent delayed effect was proposed by Martinez-Arguelles who offered that DEHP may act through a “second hit” phenomenon with initial latency [29]. Immaturity of sodium retention mechanisms of the very premature kidney is another explanation for this delay [30, 31]. The distal nephron may be unresponsive to MR stimuli until maturation of sodium transport function has occurred. Two observations support this explanation: ENaC expression in urinary exosomes, as an indirect measure of channel expression and activity, was not consistently elevated in hypertensive patients relative to controls until 40 weeks PMA; and, perhaps more significantly, pNCC was not elevated over the normotensive cohort prior to 36 weeks PMA, but manifested a higher expression thereafter until resolution of hypertension. This presumed tubular immaturity in the sodium channel and transporter expression may be analogous to that seen for neonatal potassium excretion, with a paucity of flow-dependent conducting potassium channels early in life [32].

The urine metabolite results suggest that both IV and respiratory DEHP exposures yield systemic absorption and formation of urinary MEHP. The data is not robust enough to estimate the magnitude or variability of DEHP that might leach from the various devices, nor can we predict how different respiratory DEHP exposures compare with each other or with IV DEHP exposures concerning the effect on blood pressure. Previous studies have shown a systemic transfer from DEHP-containing IV bags and PVC tubes into the bloodstream or other tissues [33, 34]. DEHP does not form covalent bonds to the PVC polymer, allowing DEHP to leach out of the PVC polymer matrix. The rate of DEHP leaching is influenced by lipophilic qualities of the fluid in contact, temperature, contact time, contact surface area, and fluid flow rate [33, 35–39]. There are estimates of DEHP leaching rates for endotracheal tubes [4], blood transfusions [40], lipid infusions [33], but not for specific PVC respiratory circuitry or IV fluid contained in bags with DEHP. One NICU unit, which labeled itself as “phthalate-free” signifying attempts to use DEHP-free equipment when available, still found detectable levels of MEHP in all of the 149 urine specimens tested [5].

Studies on mechanisms and effects of phthalate toxicity performed mostly on rats have examined both maternal and developing infant exposure [14–16, 29, 41]. Endocrine disruption effects, described as anti-androgenic, have been extensively reported from prenatal phthalate exposure and are summarized in a recent review [42]. Martinez-Arguelles et al. recently reviewed current knowledge of mechanisms mediating endocrine disruption from in-utero DEHP exposure that include alterations in adrenal steroidogenesis as well as evidence of epigenetic changes in rats leading to disease later in life [29, 41]. Studies of direct DEHP exposure in children have shown evidence of both endocrine and non-endocrine toxicity and have been reviewed elsewhere [14, 43, 44].

Aside from Zhao’s work, there are few other studies suggesting mechanisms that might explain how phthalate exposure could increase blood pressure. There is evidence that phthalates may cause oxidative stress [45, 46]. Kambia et al. treated infants and children with hyperalimentation given through PVC tubing containing DEHP and found that plasma DEHP levels correlated with malondialdehyde, a marker of free radical production [38]. Such oxidative stress may produce temporary changes in arterial tone, which increases blood pressure [47, 48]. Meanwhile, Zhao’s work, which exposed human tissue to various phthalates, convincingly demonstrated that MEHP (but not DEHP) was a potent inhibitor of human11β-HSD2 at clinically relevant concentrations [11]. When 11β-HSD2 is inhibited by black licorice, or when it is deficient as a result of genetic mutations (apparent mineralocorticoid excess), the result is sodium retention and hypertension [49].

Analysis of our data strongly suggests the mechanism of hypertension in our study cohort includes activation of the mineralocorticoid receptor. Our demonstration of increased sodium channel and transporter expression predicting higher SBP index aligns with activation of the MR, for ENaC at the height of the SBP index difference, and for pNCC throughout the entire time frame of the difference in SBP index between the two groups (PMA weeks 36 to 42). The evidence showing that urinary cortisol-to-cortisone ratio mediates the effect of IV DEHP on the SBP index suggests that inhibition of 11β-HSD2 by DEHP metabolites leads to MR activation in our cohort, in keeping with the observation by Zhao on the effect of DEHP metabolites on 11β-HSD2 [11].

The same clinical characteristics of low PRA, presentation around 35–40 weeks PMA, and an excellent response to spironolactone therapy—all seen in both our prior study [12] and in that of Al Awad et al. [13]—were also seen in this current study cohort. These similarities suggest that the mechanism of action found in this cohort may be generalizable to other premature infants with idiopathic or unexplained low-renin hypertension, with or without chronic lung disease.

Strengths of this study include its prospective design as well as urine metabolite data demonstrating DEHP transfer from medical devices to patients. Further, at least two analyses lend credence to the study’s hypotheses: (1) the linear relationship between IV exposures and SBP difference and (2) the analysis showing this relationship was mediated by urine cortisol-to-cortisone ratio. Weaknesses of the study include the lack of precise quantitation of DEHP exposures from respiratory devices and the small sample size. With only 18 subjects for analysis, we were limited in the complexity of our model building and therefore tested only the main variables of interest, which were IV and respiratory exposures to DEHP, and the effect that exposures had on SBP index and the cortisol-to-cortisone ratio. Ideally, we would have been able to control for important variables such as gestational age or similar markers of extreme prematurity previously linked to hypertension in premature infants.

In conclusion, both IV and respiratory DEHP exposures are greater in hypertensive patients as compared to normotensive patients. Cumulative IV DEHP exposure is predictive of SBP index, a relationship mediated via the cortisol-to-cortisone ratio. As DEHP is metabolized to MEHP, a known inhibitor of 11β-HSD2, a plausible mechanism of MR-mediated sodium retention, increased blood pressure, and low PRA is proposed. These results are provocative as to the potential role played by phthalates in idiopathic hypertension in premature infants. To determine the significance and safety of DEHP in respiratory tubing in regard to blood pressure effects, further studies are warranted.

## Electronic supplementary material

ESM 1 (DOCX 24 kb)

ESM 2 (DOCX 12 kb)

## Abbreviations

DEHP: Di-(2-ethylhexyl) phthalate
PVC: Polyvinyl chloride
IV: Intravenous
NICU: Newborn intensive care unit
MR: Mineralocorticoid receptor
11β-HSD2: 11β-Hydroxysteroid dehydrogenase 2
AME: Apparent mineralocorticoid excess
MEHP: Mono-(2-ethylhexyl) phthalate
SBP: Systolic blood pressure
PMA: Postmenstrual age
PRA: Plasma renin activity
MEHHP: Mono-(2-ethyl-5-hydroxyhexyl) phthalate
MEOHP: Mono-(2-ethyl-5-oxohexyl) phthalate
ENaC: Epithelial Na+ channel
pNCC: Phosphorylated (activated) Na+–Cl^−^ cotransporter
