# Supplementary material for: Phthalate-associated hypertension in premature infants: a prospective mechanistic cohort study
Source: Pediatr Nephrol. 2019 Apr 26;34(8):1413–24. doi: 10.1007/s00467-019-04244-4 (PMC6579777; doi:10.1007/s00467-019-04244-4)
Supplement: Supplementary file 1 — (DOCX 24 kb) [file 467_2019_4244_MOESM1_ESM.docx]

| **Supplemental Table I.** Exposure, blood pressure, and treatment data for normotensive and hypertensive subjects | | | | | | | | | | |
| --- | --- | --- | --- | --- | --- | --- | --- | --- | --- | --- |
|  |  |  |  |  |  |  |  |  |  |  |
| Subject | IV Fluid With DEHP | Respiratory Tubing with DEHP | Plasma Renin Activity | PMA at Date of Comparison | SBP at Date of HTN/Comparison | 95th Percentile for SBP | SBP Index | Age at HTN/Comparison | Age When Medication Discontinued | Duration Treatment of HTN |
|  | (mL) | (days) | (ng/ml/h) | (weeks) | (mmHg) | (mmHg) |  | (days) | (days) | (days) |
|  |  |  |  |  |  |  |  |  |  |  |
| N1 | 0 | 4 | 2.2 | 46.86 | 77 | 85 | 0.91 | 108 | NA | NA |
| N2 | 0 | 0 | Not done | 40.14 | 94 | 95 | 0.99 | 28 | NA | NA |
| N3 | 181.3 | 0 | Not done | 36.00 | 69 | 87 | 0.79 | 8 | NA | NA |
| N4 | 0 | 0 | Not done | 36.00 | 71 | 87 | 0.82 | 8 | NA | NA |
| N5 | 0 | 5 | 1.03 | 42.57 | 76 | 98 | 0.78 | 61 | NA | NA |
| N6 | 24.8 | 23 | 47.8 | 41.57 | 76 | 98 | 0.78 | 89 | NA | NA |
| N7 | 0 | 7 | 2.9 | 39.86 | 84 | 95 | 0.88 | 53 | NA | NA |
| N8 | 0 | 3 | 10.6 | 39.86 | 84 | 95 | 0.88 | 53 | NA | NA |
| N9 | 0 | 83 | 0.3 | 39.43 | 79 | 95 | 0.83 | 84 | NA | NA |
| Mean |  |  |  | 40.25 | 79 |  | 0.85 | 54.67 |  |  |
| SD |  |  |  | 3.32 | 7.6 |  | 0.07 | 35.35 |  |  |
| Median (IR)* | 0 (0) | 7 (7) |  |  |  |  |  |  |  |  |
| H1 | 0 | 26 | < 0.2 | 35.57 | 104 | 87 | 1.20 | 48 | 193 | 145 |
| H2 | 0 | 64 | < 0.2 | 36.42 | 91 | 87 | 1.05 | 64 | NA | NA |
| H3 | 285.6 | 57 | 0.41 | 39.00 | 95 | 94 | 1.01 | 90 | NA | NA |
| H4 | 374.6 | 0 | 1.51 | 45.85 | 121 | 105 | 1.26 | 89 | 211 | 122 |
| H5 | 201.3 | 45 | 0.41 | 43.43 | 109 | 105 | 1.04 | 95 | 152 | 57 |
| H6 | 766.9 | 7 | 0.12 | 41.57 | 121 | 98 | 1.23 | 82 | 167 | 85 |
| H7 | 291.7 | 67 | 0.04 | 39.00 | 96 | 94 | 1.02 | 88 | 152 | 64 |
| H8 | 33.9 | 42 | < 0.1 | 42.71 | 106 | 98 | 1.08 | 103 | 208 | 105 |
| H9 | 0 | 75 | 0.1 | 41.72 | 104 | 98 | 1.06 | 100 | NA | NA |
| Mean |  |  |  | 40.59 | 105 |  | 1.11 | 84.33 | 180.49 | 96.33 |
| STDEV |  |  |  | 3.36 | 10.7 |  | 0.98 | 17.72 | 27.02 | 34.13 |
| Median (IR)* | 201.3 (291.7) | 45 (38) |  |  |  |  |  |  |  |  |
| *t* test | 0.040 | 0.037 |  | 0.836 | 0.000 |  | 0.000 | 0.039 |  |  |
| Wilcoxon rank sum test | 0.029 | 0.041 |  | 0.930 |  |  | < .001 |  |  |  |

N, Normotensive; H, Hypertensive; DEHP, di-(2-ethylhexyl) phthalate; PMA, Postmenstral age; SBP, Systolic blood pressure; HTN, Hypertension; SD, Standard deviation. * IV fluid with DEHP and Respiratory Tubing with DEHP are reported as median (interquartile range).
