# Supplementary material for: Phthalate-associated hypertension in premature infants: a prospective mechanistic cohort study
Source: Pediatr Nephrol. 2019 Apr 26;34(8):1413–24. doi: 10.1007/s00467-019-04244-4 (PMC6579777; doi:10.1007/s00467-019-04244-4)
Supplement: Supplementary file 2 — (DOCX 12 kb) [file 467_2019_4244_MOESM2_ESM.docx]

Isolation of Urinary Exosomes

Approximately 1-10 mL of urine was collected and treated with 1 cOmplete Mini Protease Inhibitor Cocktail tablet (Sigma) and 1 PhosSTOP Phosphatase Inhibitor (Roche). Samples were frozen at -80 degrees Celsius. The isolation procedure described by Nils van der Lubbe et al (Hypertension. 2012 Sep; 60(3):741-8) was followed except that volumes of added reagents were scaled down to 1/3 of the volumes given.

Western Analysis of Exosome Preps

Exosome pellets were re-suspended in 50uL 1.5X Laemmli electrophoresis sample buffer and heated to 65 degrees Celcius for 15 minutes. 40uL of each sample was loaded onto BioRad 4-15% Tris-glycine precast gels with 50uL wells. After electrophoresis, samples were transferred to Immun-Blot PVDF membrane (BioRad) overnight at 4 degrees Celsius. Membranes were blocked in 1xPBS containing 0.5% Tween 20 and 5% nonfat milk and were probed overnight at 4 degrees Celsius first with rabbit polyclonal anti-gamma ENaC (Stressmarq, 1:1000 in block) followed by 3 washes with block. The membranes were then incubated in a 1:5000 dilution of goat anti-rabbit IgG:HRP secondary (Santa Cruz) at room temperature for 1 hour, followed by 3 washes in 1xPBS plus Tween. Following detection with anti-gamma ENaC, the membrane was stripped with acidic membrane-stripping buffer (25mM glycine, 1% SDS pH 2.0) and re-probed overnight at 4 degrees Celsius with rabbit polyclonal anti-pNCC (1:2000 in block), courtesy of David Ellison. Secondary antibody for the anti-pNCC Westerns was prepared and used as described for the anti ENaC Westerns. Following detection with anti-pNCC, the membranes were stripped as above and re-probed with rabbit monoclonal anti CD9 (D801A clone from Cell Signaling) at a 1:1000 dilution in block overnight at 4 degrees Celsius. Secondary antibody for the anti-CD9 Westerns was prepared and used as described for the anti ENaC Westerns. All Westerns were developed with Western Lightning Chemiluminescent kit (Perkin Elmer) and imaged on a Syngene Pxi imager. Syngene software was used to call bands and generate densitometry data.
